# High-glucose diets have sex-specific effects on aging in *C. elegans*: toxic to hermaphrodites but beneficial to males

**DOI:** 10.18632/aging.100759

**Published:** 2015-06-08

**Authors:** Marjorie R. Liggett, Michael J. Hoy, Michae Mastroianni, Michelle A. Mondoux

**Affiliations:** ^1^ Department of Biology, College of the Holy Cross, Worcester, MA 01610, USA

**Keywords:** glucose, C. elegans, sex specificity, mobility, healthspan

## Abstract

Diet and sex are important determinants of lifespan. In humans, high sugar diets, obesity, and type 2 diabetes correlate with decreased lifespan, and females generally live longer than males. The nematode *Caenorhabditis elegans* is a classical model for aging studies, and has also proven useful for characterizing the response to high‐glucose diets. However, studies on male animals are lacking. We found a surprising dichotomy: glucose regulates lifespan and aging in a sex‐specific manner, with beneficial effects on males compared to toxic effects on hermaphrodites. High‐glucose diet resulted in greater mobility with age for males, along with a modest increase in median lifespan. In contrast, high‐glucose diets decrease both lifespan and mobility for hermaphrodites. Understanding sex‐specific responses to high‐glucose diets will be important for determining which evolutionarily conserved glucose‐responsive pathways that regulate aging are “universal” and which are likely to be cell‐type or sex‐specific.

## INTRODUCTION

Diet is a key regulator of lifespan: caloric intake, and in particular, glucose metabolism, must be tightly controlled for health and survival. High levels of glucose lead to toxicity, which can manifest in humans as obesity and type 2 diabetes. These diseases, in turn, are correlated with decreased fertility and lifespan. Given the global rise in sugar consumption, understanding glucose toxicity and its role in health and aging is a crucial public health problem [1]. *Caenorhabditis elegans* is an excellent model for studying aging and glucose toxicity, as worms have a well-conserved insulin-signaling system implicated in both processes. Mutations that decrease insulin signaling extend lifespan [2], and insulin signaling is up regulated in response to excess glucose [3, 4]. As in humans, excess glucose leads to toxicity in *C. elegans*: high-glucose diets lead to decreased fertility and lifespan [3-7]. In addition to differences in lifespan, differences in aging can be assessed by measuring healthspan, the period before age-related decline when animals maintain at least 50% of their functional capacity [8, 9]. High-glucose diet leads to a reduction in functional capacity in *C. elegans* by several measures, including the locomotion response to touch [10], the ability to survive oxygen deprivation [11], the structural integrity of the nervous system [12], and pharyngeal pumping [4]. Declines in some healthspan parameters, like pharyngeal pumping, have been shown to correlate with declining lifespan [13]. High-glucose diet reduces many of these healthspan parameters even in young adults, indicating that glucose affects age-associated phenotypes long before differences in lifespan potential can be observed [4, 11, 12].

Sex is also a key regulator of lifespan: different sexes have different average lifespans in a wide range of species, including humans. *C. elegans* exists in two sexes: males and self-fertilizing hermaphrodites, which anatomically resemble females that store sperm. *C. elegans* hermaphrodites and males are distinct from each other in anatomy, physiology, behavior, and gene expression [14]. Lifespan in *C. elegans* males, but not hermaphrodites, is regulated by social interactions with hermaphrodites and other males [15]. Despite these differences, most experiments in this model system, including the studies on high-glucose diet and aging, have been conducted exclusively with hermaphrodites.

Here, we tested the effects of a high-glucose diet on *C. elegans* lifespan and aging in both sexes. Surprisingly, we found that a high-glucose diet produced a diametric aging response in *C. elegans*: toxic to one sex (heramphrodites) but beneficial to the other (males). In contrast to its well-established negative effects on hermaphrodites, a high-glucose diet was not toxic to males. Rather, high-glucose diet specifically protected male mobility with age and produced a modest lifespan extension in males.

## RESULTS

**Figure 1:**
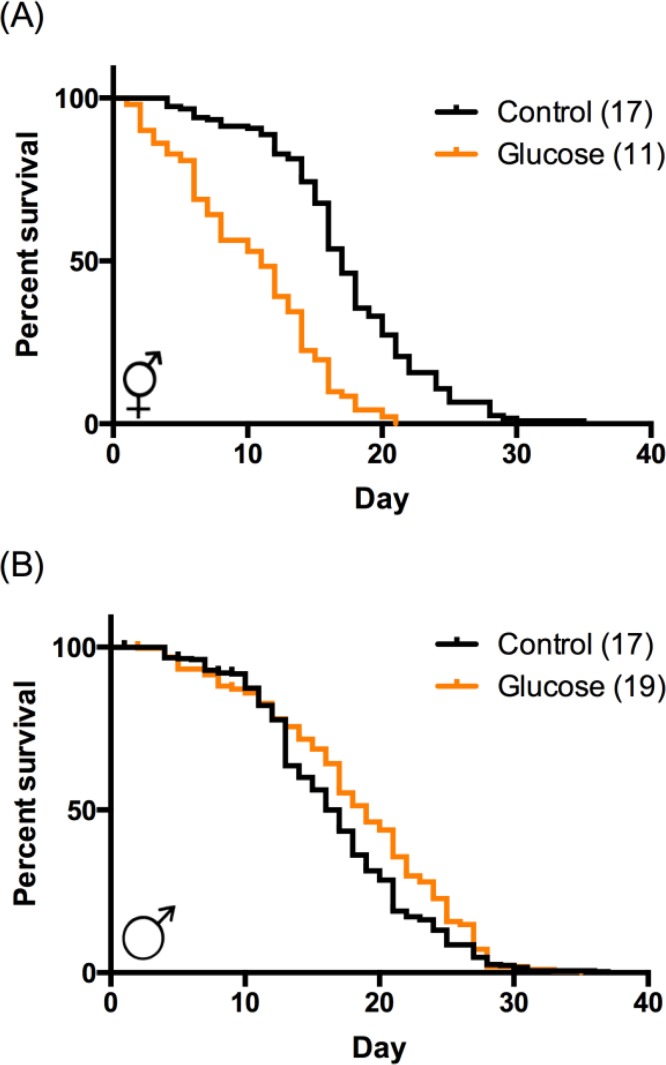
High‐glucose diet regulates lifespan in a sex‐specific manner. (**A**) In hermaphrodites, high‐glucose diet (orange) reduced median and maximum lifespan compared to control (black). p < 0.0001 by log‐rank. (**B**) In males, high‐glucose diet increased median lifespan (p < 0.0001 by log‐rank) and had no effect on maximum lifespan. Median lifespans are indicated in parentheses. Composites of at least 3 replicates are shown. Individual experimental data are provided in Table S1.

We tested the effects of a high-glucose diet on two aging parameters in *C. elegans*: lifespan and mobility, which is an important measure of healthspan. Consistent with previously published reports, high-glucose diet significantly decreased both median and maximum hermaphrodite lifespan by 30-40% (Fig. 1A; [3, 6, 7]). In contrast, high-glucose diet did not decrease maximum lifespan in males (Fig. 1B), suggesting that the negative effects of glucose on lifespan are sex-specific. Indeed, high-glucose diet modestly increased average longevity (10% increase; Fig. 1B), indicating a sexually diametric response. *C. elegans,* like humans, demonstrate a decline in both coordination and mobility with age, in concert with loss of muscle mass, or sarcopenia [9, 13, 16, 17]. In hermaphrodites, this decrease in mobility is regulated by insulin signaling [17] and in males by the NAD+-dependent metabolic regulator SIR-2.1 [18], suggesting a potential role for glucose in this process. Indeed, hermaphrodites aged on high-glucose diet have decreased locomotion in response to touch [10]. Mobility is an especially important measure of healthspan for males, as their ability to reproduce depends on their ability to seek mates and perform mating behaviors, which also undergo an age-related decline [19].

**Figure 2:**
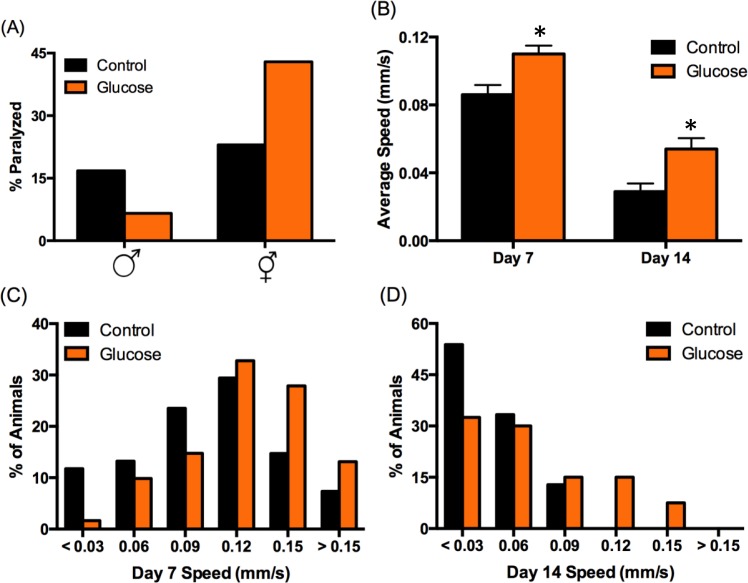
High‐glucose diet regulates mobility in a sex‐specific manner. (**A**) High‐glucose diet (orange bars) decreased male paralysis (increased mobility) and increased hermaphrodite paralysis (decreased mobility) compared to control (black bars) at day 14 of adulthood. (**B‐D**) Male mobility was analyzed using Multi‐Worm Tracker. **(B)** Average speed decreases with age on both diets (p < 0.001 by ANOVA), but average speed on high‐ glucose diet was significantly higher than control on day 7 and day 14 (*p < 0.001 by ANOVA). Error bars represent SEM. **(C‐D)** The distribution of observed speeds was significantly different for males on high‐glucose diet compared to control on day 7 **(C)** and day 14 **(D)** (p = 0.02 and 0.004, respectively, by Kolmogorov‐Smirnov Z test).

We observed visually striking, sex-specific differences in mobility of animals aged on high-glucose diet compared to control. We quantified these changes by assaying paralysis at day 14 of adulthood, when the effect of glucose on male lifespan becomes apparent (Fig. 1B). On control diet, both sexes had similar levels of paralysis in the population (17% male, 23% hermaphrodite; Fig. 2A). Consistent with previous reports of glucose reducing mobility [10] and other healthspan parameters [4, 11, 12], high-glucose diet increased paralysis in the aged hermaphrodite population by almost twofold (43%). In contrast, high-glucose diet reduced paralysis in the male population 2.4-fold (7%; Fig. 2A), suggesting that a high-glucose diet might prevent age-related mobility decline specifically in males.

We further analyzed male mobility by tracking speed. Glucose-induced changes in mobility were observed at day 7 of adulthood, which is just post-reproductive [20] and is before discernable lifespan effects (Fig. 1B). Males on high-glucose diet moved ~25% faster on day 7 compared to control (Fig. 2B), indicating that diet influences healthspan before lifespan. Consistent with our paralysis data (Fig. 2A), although mobility declined with age on both diets, males aged on high glucose were significantly more mobile on day 14 of adulthood, moving ~85% faster than control (Fig. 2B).

At any given age in a population, there is a range of healthspan phenotypes, including varying mobility, which may be predictive of how long an individual animal will live [13, 21]. A wide range of speeds was observed on both diets at both 7 and 14 days of age, from paralyzed animals to animals that move quite rapidly. However, high-glucose diet reduced the number of paralyzed or extremely slow-moving males by ~85% on day 7 and ~40% on day 14 (Figs. 2C-D). Likewise, nearly twice as many 7-day old males on high-glucose diet reached the fastest speeds compared to control (> 0.12 mm/s; Fig. 2C). This increase in fast-moving animals was further pronounced on day 14, as none of the control males reached speeds above 0.09 mm/s, compared to 23% of the animals on high glucose (Fig. 2D). These data demonstrate that high-glucose diet improves an important measure of healthspan in *C. elegans* males, in contrast to its effects on *C. elegans* hermaphrodites (Fig. 2A; [4, 10–12]).

## DISCUSSION

Why is glucose toxic to hermaphrodites but beneficial for male aging? There is evidence that the relationship between lifespan and diet is sex-regulated. Dietary restriction (DR) extends lifespan in many species. Some of the effects of DR in *C. elegans* (*e.g*., body size) are more pronounced in hermaphrodites compared to males, and DR differentially regulates several hundred genes between the sexes [22]. Furthermore, rapamycin, which partially mimics DR, has a more potent lifespan extension effect on female mice compared to males [23], and lifespan is maximized under different DR conditions in *Drosophila* females compared to males [24].

Since glucose occupies a central role in metabolism, there are seemingly limitless, non-mutually exclusive ways it could regulate aging in a sex-specific manner. Over 2,000 genes have been identified as differentially expressed in response to a high-glucose diet in *C. elegans* hermaphrodites [3, 11], and nearly an equal number are differentially expressed between males and hermaphrodites on a control diet [25]. Any of these genes may be involved in the sex-specific response to glucose. Potential mechanisms include sex-specific differences in carbohydrate storage [26], sex-specific regulation of pathways that respond to glucose or insulin signaling [15, 27], the role of the reproductive system in regulating lifespan [28], differences in glucose usage and processing in sex-specific cells (males have 89 additional neurons and 41 additional muscle cells [29, 30]), and so on.

We have identified high-glucose diet as a factor that specifically protects mobility during aging in male *C. elegans*. This increase in healthspan occurs without a concomitant extension in maximum lifespan. In humans, where advances in health care have led to longer median, but not maximum, lifespans, this extension of healthspan is termed “compression of morbidity” or “healthy aging”. Recently, it has been suggested that lifespan be divided into two parts: healthspan, in which animals have 50% or greater functional capacity, and “gerospan”, when functional capacity declines below 50% [9]. A key goal for aging research, then, is to extend healthspan without extending gerospan, as we show here for a high-glucose diet in male *C. elegans*.

Although other mutations and dietary interventions have been shown to have sex-specific lifespan phenotypes in *C. elegans* [15, 22], these differences have been a matter of degree (*i.e.* the effect is greater in one sex compared to the other or affects one sex and not the other). Uniquely, high-glucose diets produce a sexually diametric aging response: beneficial to one sex (males) and toxic to the other (hermaphrodites). Recently, the National Institutes of Health (NIH) called for balancing sex in preclinical studies to increase the inclusion of females [31]. In *C. elegans,* the male has often been excluded from study. The unique sex-specific response to high-glucose diet identified here underscores the need to study the response to dietary interventions in both sexes in order to maximize the usefulness of *C. elegans* as a model for human aging and metabolic disease.

## METHODS

### Maintenance and strains

All of the experiments described used the wild type N2 (Bristol) strain of *C. elegans* maintained at 20**°**C on solid Nematode Growth Medium (NGM) using *E. coli* OP50 as a food source. Males were maintained using small mating plates with 7-8 males and 2 adult hermaphrodites. All animals were allowed to develop on NGM control plates. High glucose feeding began in the late L4 stage on NGM plates supplemented with 250 mM glucose.

### Lifespan assays

Populations were synchronized by hypochlorite treatment to isolate embryos followed by visual selection of late L4 animals. Hermaphrodites were grouped 5-10 animals per plate (as in [3]) and transferred to new plates every 1-2 days until reproduction ceased (~7 days). Males were maintained on individual plates to avoid male-male interactions that influence lifespan; hermaphrodite lifespan was not affected by singling animals ([15] and data not shown). Animals were scored as dead if they did not respond to gentle prodding with a worm pick. Animals that died because of bagging or crawling off the plate were censored. GraphPad Prism v. 6 was used for analysis and *P* values were calculated using the log-rank test.

### Mobility assays

Animals were aged on control or high-glucose diet as described above and tested for paralysis at day 14 of adulthood. Animals were scored as paralyzed if they moved only their heads in response to prodding with a worm pick. n = 60-200 animals per condition. Male speed was quantified on days 7 and 14 of adulthood using a Pike F421b camera from Allied Vision Technologies and the Multi-Worm Tracker video recording and Choreography analysis software [32]. Animals were recorded individually for 60 seconds. Only forward motion lasting at least 10 seconds and covering at least one body length was used to calculate average speed, and only one average speed was calculated per animal. Animals that did not move forward at least one body length during the time course were counted as paralyzed (speed = 0 mm/s). Animals used for analysis with the Worm Tracker were not returned to the population. n = 40-70 animals per condition. *P* values were calculated using a two-way analysis of variance (ANOVA) for mean speeds and a two-tailed Kolmogorov-Smirnov Z test for speed distributions.

## SUPPLEMENTARY TABLE


