# Supplementary material for: High-glucose diets have sex-specific effects on aging in C. elegans: toxic to hermaphrodites but beneficial to males
Source: Aging (Albany NY). 2015 Jun 8;7(6):383–7. doi: 10.18632/aging.100759 (PMC4505165; doi:10.18632/aging.100759)
Supplement: Supplementary file 1 [file aging-07-0383-s001.pdf]

SUPPLEMENTARY TABLE

Table S1. Lifespans shown in Figures 1A and 1B.
20°C

Sex	Diet	Median lifespan (days)	Maximum lifespan (days)	No. animals	Median lifespan change	Maximum lifespan change	*P* value against control	No. of Exp.	Figure
Hermaphrodite	Control	17	36	128/151	N/A	N/A	N/A	3	1A
	Glucose	11	22	149/151	-35%	-39%	< 0.0001	3	1A
Male	Control	17	36	353/369	N/A	N/A	N/A	8	1B
	Glucose	19	36	320/371	+10%	0%	< 0.0001	8	1B

Data shown are a composite of 3 or 8 individual experiments, which are described individually below. Synchronized late L4 adults were transferred to lifespan plates (control NGM or NGM plus 250 mM glucose). *P* values were obtained by log-rank. Median and maximum lifespan were shorter on glucose compared to control in hermaphrodites. Median lifespan was longer, and maximum lifespan unchanged, on glucose compared to control in males.

Sex	Diet	Median lifespan (days)	Maximum lifespan (days)	No. animals	Median lifespan change	Maximum lifespan change	*P* value against Control	Assay #
Hermaphrodite	Control	17	31	51/51	N/A	N/A	N/A	1
	Glucose	8	21	51/51	-53%	-32%	< 0.0001	1
	Control	17	25	35/50	N/A	N/A	N/A	2
	Glucose	12	20	48/50	-29%	-20%	< 0.0001	2
	Control	16	36	42/50	N/A	N/A	N/A	3
	Glucose	12	22	50/50	-25%	-39%	< 0.0001	3
Male	Control	17	33	50/50	N/A	N/A	N/A	1
	Glucose	18	29	47/50	+6%	-12%	0.8	1
	Control	11	36	45/50	N/A	N/A	N/A	2
	Glucose	17	31	47/50	+55%	-14%	0.2	2
	Control	18	31	49/50	N/A	N/A	N/A	3
	Glucose	16	29	46/51	-11%	-6%	0.7	3
	Control	17	23	19/20	N/A	N/A	N/A	4
	Glucose	20	23	20/20	+18%	0%	0.09	4
	Control	15	28	49/50	N/A	N/A	N/A	5
	Glucose	21	28	42/50	+40%	0%	<0.002	5
	Control	21	34	47/50	N/A	N/A	N/A	6
	Glucose	22	34	39/50	+5%	0%	0.9	6
	Control	13	25	45/50	N/A	N/A	N/A	7
	Glucose	20.5	36	30/50	+58%	+36%	<0.0001	7
	Control	16	23	49/49	N/A	N/A	N/A	8
	Glucose	16	27	49/50	0%	+17%	<0.02	8

Assay numbers indicate trials that were performed in parallel. For hermaphrodites, median lifespan on high-glucose diet was shorter than median lifespan on control diet in each trial (3/3), and maximum lifespan was shorter on glucose compared to control for each trial (3/3). For males, median lifespan on high-glucose diet was longer than (6/8) or equal to (1/8) median lifespan on control diet in all but one trial. High-glucose diet had no overall effect on maximum lifespan in males: in some trials maximum lifespan was equal (3/8), and in others it was longer (2/8) or shorter (3/8) on glucose compared to control.
